# Bisphosphonates and implants: An overview

**DOI:** 10.1080/17453670902807466

**Published:** 2009-02-01

**Authors:** Per Aspenberg

**Affiliations:** ^1^Orthopaedics Section, Department of Clinical and Experimental Medicine, Linköping UniversityLinköpingSweden

## Background

Bisphosphonates selectively inactivate osteoclasts. In some situations they can do this while osteoblast activity is maintained. This leads to increased net bone formation.

**Figure 1.**
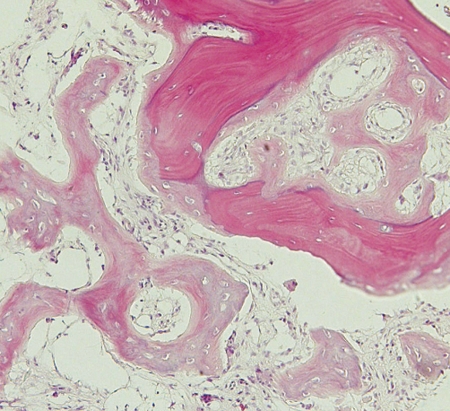
Biopsy from distal radial fracture 19 days after injury. An old, partly necrotic trabecula and woven bone forming without cartilage precursor within the marrow space.

**Figure 2.**
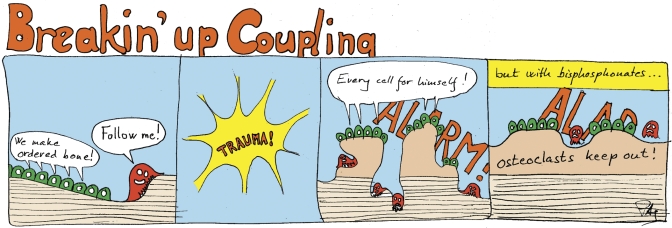
Bone resorption and formation are coupled during remodeling, but not during the response to trauma.

Bisphosphonates are powerful drugs. Their ability to reduce the risk of osteoporotic fractures has saved thousands of people from becoming a fracture patient. Osteoporosis is due to an imbalance in the maintenance remodeling of the skeleton. This remodeling is different from the repair processes following fracture or bone surgery. Because research on the use of bisphosphonates has mostly focused on osteoporosis, their effects on repair processes have long been overlooked. In bone remodeling, osteoclasts and osteoblasts work closely together—also in a spatial sense—at defined remodeling sites. Their activities are coupled: a decrease in bone resorption due to bisphosphonates leads to a reduction in bone formation to a similar degree. Thus, it was long thought that bisphosphonates would only slow down bone repair. However, in bone repair, osteoblasts can work independently. This is clear if you just look at it in the microscope, where you can see large areas of undisturbed bone formation, separate from areas undergoing resorption and remodeling (Figure 1). A reduction in osteoclast activity can therefore be expected to shift the balance between formation and resorption towards increased net bone formation. During bone repair, bisphosphonates have an anti-catabolic, or net anabolic, effect (Figure 2) (Little et al. 2005, Wermelin et al. 2007).

Bisphosphonates bind to bone mineral and are taken up by osteoclasts when the latter resorb bone, which inactivates the cell. This mechanism makes bisphosphonates highly osteoclast-specific; free bisphosphonate is quickly excreted via the kidneys. However, some macrophages may be affected, and there is in vitro evidence (Garcia-Moreno et al. 1998, Giuliani et al. 1998) that osteoblasts are stimulated by concentrations of bisphosphonates that are unlikely to occur in vivo (Schindeler and Little 2005).

It has been suggested that bisphosphonates may impair fracture healing. Osteoporotic fractures indicate an increased risk of new fractures, and should be an incentive to start prophylactic bisphosphonate treatment. Now, if bisphosphonates impair fracture healing, it would be wise to delay the start of secondary prevention until the fracture is healed. In animal experiments, bisphosphonates increase fracture callus size and delay the regression in size after the fracture has healed (Madsen et al. 1998, Cao et al. 2002). No mechanical impairment had been demonstrated. In large clinical series, no increase in healing complications has been reported (Lyles et al. 2007). It would therefore seem free of risk to start bisphosphonates directly after the first osteoporotic fracture. However, if intravenous bisphosphonates are used, there is a risk that the affinity of the fracture site for bisphosphonates would be so large that the rest of the skeleton would not be reached by a sufficient amount, and remain untreated (Lyles et al. 2007).

The aim of this article is to discuss bisphosphonates together with implants in bone. I therefore now leave osteoporosis treatment.

## Fracture fixation devices

Systemic bisphosphonate treatment can increase screw removal resistance. Screws can also be better fixated in bone if they are coated with bisphosphonates.

Plates and marrow nails have low friction to the bone, and transfer forces via large surfaces that lie on to bone and take up compressive forces. Their function is entirely due to gross shape. Screws are different: unlike nails, their conformation allows them to take up forces in all directions, except in rotation around their axis. Complex fractures need screws. The strength of screw fixation depends on the strength of the bone close to the threads. For pull-out forces, this bone acts as a screw nut. In cortical bone, this screw nut is threaded at insertion, and can have considerable strength. This may decrease with time, as the cortical bone is likely to resorb in response to the trauma (Dhert et al. 1998). In cancellous and osteoporotic bone, however, trabeculae might fracture at a distance from the drill or screw thread, making the “screw nut” even weaker than the intact cancellous bone would suggest. On the other hand, cancellous bone has a remarkable capacity to regenerate and will start making new bone at the traumatized site, and eventually form a bone shell around the screw. So whereas screw fixation in cortical bone is strong, with a risk of slight decrease over time, cancellous bone is weak with a tendency to gradual improvement. Unfortunately, there is often no time to wait for such improvement.

**Figure 3.**
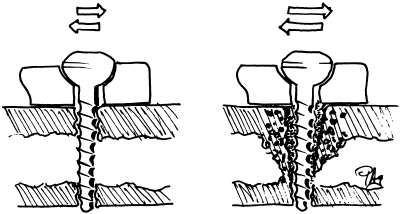
The observation that microinstability of a screw may lead to resorption and gross loosening may explain the role of local fluid flow in bone resorption. Forces and hydrostatic pressure around a microunstable screw are not greater than around a fixed one, but local fluid flows are. Drawn from memory of a drawing by Perren.

Most patients are old, and unlike the young, they have little time. Our common fracture patients need to come back to load bearing quickly. If this does not lead to immediate mechanical failure of the fracture fixation, there is risk of microinstability, leading to bone resorption around the screws (Ganz et al. 1975) (Figure 3) or accumulated damage leading to collapse later on. There will be a race between these destructive processes and the repair response initiated by screw insertion trauma (and the fracture). Any therapy that would favor bone formation and inhibit bone loss around the screw would buy time and increase the chance that the fixation construct would last until the fracture is healed. This is where bisphosphonates come in.

In one randomized clinical study, hydroxyapatite-coated external fixation pins showed a higher removal torque in patients given a bisphophonate systemically (Moroni et al. 2007). In another randomized study, the same was found for metal pins—but not when they were coated with hydroxyapatite (Tägil et al. 2008). For local treatment, and possibly a stronger effect, bisphophonates can be injected directly into the screw hole. This has resulted in improved screw fixation in rats (Skoglund et al. 2004), but in clinical situations the bisphosphonate solution is likely to be flushed away by bleeding and the treatment would be difficult to control. This problem could be overcome by specific devices such as a bisphosphonate-soaked foam plug inserted in the screw hole followed by an expander, which squeezes the solution out of the foam into the surrounding bone. Although feasible, this would be less practical than simply putting the bisphosphonate directly on the screw surface. This can be done by several techniques, which have been evaluated in animal experiments.

Most experiments use calcium phosphate coatings (often hydroxyapatite) as a base (Peter et al. 2005, 2006). The test implants are more often rods than screws. They just need to be placed in a bisphosphonate solution and the affinity will adsorb the bisphosphonate to the calcium phosphate. Several groups have shown improved fixation with such treatments. One of them reported a risk of overdosing with amounts of bisphosphonate on the surface that are too high (Peter et al. 2005). However, the number of animals was small, and these overdose effects were not reproduced in a similar experiment with more precise evaluation (Peter et al. 2006). Others have used a polylactic-glycolic acid polymer for slow release of the bisphosphonate (Greiner et al. 2007).

The group of Pentti Tengvall and myself has used a crosslinked fibrinogen layer, covalently linked to metals via silanes. The crosslinked fibrinogen can bind several bisphosphonates chemically (Tengvall et al. 2004). This fibrinogen coating can be produced by simple chemical methods and can be sterilized by radiation (Wermelin et al. 2008b). Although fibrinogen is a protein, the crosslinking is so tight that it is more to be regarded as a polymer. The bisphosphonate appears to be released over a period of hours or days and the fibrinogen is degraded in a matter of weeks. The effects we have seen in a rat model are extraordinary. The pullout force for stainless steel screws was increased compared to controls after 2 weeks. More remarkably, however, this difference increased continuously over time, so that the pullout force was doubled after 8 weeks. Histology showed that a shell of new bone had formed around the screw, inside the marrow cavity—something that was not seen in the controls (Wermelin et al. 2008a, b). This does not mean that bisphosphonates induce new bone formation like, for example, a BMP. The explanation appears to be that very scant, patchy loose bone or osteoid formed also around the control screws after 1 week, but then disappeared when resorption was not suppressed. When protected by the bisphosphonate, this early, scant bone served as a scaffold for continued bone growth, leading to the formation of a bony shell.

## Joint replacements

The initial stability of joint replacements can be improved both by systemic and local bisphosphonates.

The initial fixation of joint prostheses appears to be crucial for long-term success (Kärrholm et al. 1994, Ryd et al. 1995). The surfaces of joint replacements are mostly adjacent to cancellous bone. There is often a gradual change in position of the prosthesis during the first weeks, which can be explained by microfracturing, until the prosthesis has settled into a position in which the load is evenly distributed to the bone. This is not the only explanation, however. By use of radiostereometry, we have shown that both systemically and locally applied bisphosphonates reduce this early migration (Hilding et al. 2000, Hilding and Aspenberg 2006, 2007). This means that osteoclasts are involved. The bisphosphonates may inhibit resorption of the bone next to the prosthesis, which can be nothing but necrotic and prone to resorption. Another possibility is that the response to the trauma gets a more positive balance between bone formation and resorption, as previously described for screws. The first study used a rather high dose of clodronate given systemically, starting 3 weeks before surgery (Hilding et al. 2000). The second used ibandronate applied locally to the cut bone surface immediately before cementation (Hilding and Aspenberg 2007).

A third knee study failed to show any effect of a bisphosphonate upon radiostereometric migration. In contrast to the other 2 studies, this study used an uncemented prosthesis and a conventional osteoporosis-based dose of alendronate taken orally (Hansson et al. 2009). The reason that it did not work may be that uncemented prostheses migrate much more than cemented ones. This probably represents settling due to unevenness of the cut bone surfaces. At first, only small cancellous areas have to take all the load, and they are likely to be compacted. Any effect upon resorption might be hidden in the variation associated with this larger migration. Another possibility might be that the dose of alendronate was too low.

The early migration of hip prostheses can be reduced by bisphosphonates. In a randomized double-blind study, a single infusion of zoledronate abolished the migration of the cup and improved the Harris hip score (Friedl et al. 2009). The study used a less precise method than RSA, and another similar study showed no effect (Wilkinson et al. 2005). It has recently been shown that zoledronate given as a single intravenous infusion after hip surgery will to a great extent be concentrated to the traumatized bone region, so that local treatment may not be necessary to achieve a high local dose (Amanat et al. 2007).

The 2 positive knee studies both showed a reduction in “maximal total point motion” from about 0.4 to 0.3 mm at 1 year. How can this small difference be of any clinical importance? There are three reasons to believe this. First, migration is associated with microinstability, which indicates that there is a fibrous membrane between the implant and the bone (Hilding et al. 1995). This membrane allows generation of local fluid flow and gives access to particulate debris, both of which are known to induce bone resorption and thereby loosening.

Secondly, there has been a study on 155 knee prostheses of various types, which were included in different studies and then analyzed retrospectively when 12 of them had been revised for loosening (Ryd et al. 1995). It turned out that those that loosened had migrated statistically significantly more already at 6 months. The highest predictive power was found for migration during the second year. This study investigated patients who were operated in the 1980s, however, when the quality of implants and surgery was still developing. Both the failure rate and the migration were higher than in later years. The study would probably be impossible to repeat, because the low failure rate with modern methods would necessitate an impossibly high number of patients to achieve a reasonable statistical power.

A third reason to believe that early migration is ominous would be if there is a dichotomy between stable and migrating prostheses (or between almost stable prostheses and those with more migration). We have recently shown that such a dichotomy exists for cemented acetabular cups (Aspenberg et al. 2008). Most cups did not migrate at all: their radiostereometric values apparently represented signal noise. Some cups, however, showed true migration. Preliminary data from my group indicate that there is a similar dichotomy for both cemented and uncemented tibial components (unpublished observations). This indicates that the small reduction in mean value induced by the bisphosphonates (0.3 mm as opposed to 0.4 mm) in reality represents a large reduction in the number of migrating implants. If we believe that lack of stable fixation puts the patient at risk of loosening, bisphosphonate treatment would be a good idea.

Several studies have shown that bisphosphonates can prevent the loss in bone density normally seen at some distance from prostheses by DXA, especially bone loss due to “stress shielding”. Although a striking effect, it is uncertain whether this has any relevance for prosthetic fixation (Bhandari et al. 2005).

Can bisphosphonates be used to stop the progress of symptomatic loosening? Attempts to show this in a large randomized study have, to my knowledge, failed and remained unpublished. We have also tried local injection of bisphosphonates into the pseudojoint. Because this communicates with the space around the loose prosthesis (the “effective joint space”), we hoped to expose the periprosthetic bone to a higher concentration. In a few pilot cases, this did not seem to have any effect. We therefore injected a radioactive technetium-labeled bisphosphonate and could see on a scintimetric tomogram that the bisphosphonate was taken up by the general circulation from the loosening membrane before reaching the bone (unpublished observations).

The apparent lack of an effect of systemic treatment for blocking progress of loosening can be explained by the fact that bisphosphonates never inhibit resorption completely; osteoclasts must resorb some bone in order to be intoxicated by the bisphosphonate. If the resorptive stimulus is strong, and the number of recruited osteoclasts is high, the bone will be resorbed anyway (Åstrand and Aspenberg 2002). However, drugs that block the RANKL signaling system, such as osteoprotegerin or monoclonal antibodies to RANK, may be more potent for this indication because they inhibit osteoclast recruitment instead of reducing their activity. This might lead to complete inhibition of resorption.

## Dental implants

Dental implants need time to osseointegrate before loading. This time might be reduced by local bisphosphonate treatment.

Dental implants are screws inserted in the jawbones. Normally, they are left unloaded after insertion, hidden under the gingiva. Months later, when they have osseointegrated, they are re-exposed and tooth replacements are attached to them. As bisphosphonates accelerate mechanical fixation under other clinical and experimental conditions, they might shorten the waiting time until loading of dental implants. We have made a pilot study in which 5 edentolous patients received 7 dental implants each, 1 of which was coated with bisphosphonates. Stability of fixation was estimated by measuring vibration resonance frequency. In all 5 patients, the bisphosphonate-coated implants showed the greatest improvement in resonance frequency (unpublished observations). This study was not randomized or blinded, and there are feasible explanations for the findings other than that of a bisphosphonate effect. Still, the results suggest that it may be worthwhile to study whether bisphosphonate-coated implants can reduce the time until loading for dental implants. It is a problem, however, that the condition known as “osteonecrosis of the jaw” has caused fear of using bisphosphonates in conjunction with oral surgery. This condition is not really an osteonecrosis, but an osteomyelitis, in which bisphosphonate-containing bone probably cannot be resorbed fast enough for the lesion to heal (Aspenberg 2006, Dodson et al. 2008). At coated dental implants only the bone adjacent to the implant would contain bisphosphonate, and it could easily be removed together with the implant, if needed.

## Conclusion

Although antiresorptive, bisphosphonates may paradoxically increase the amount of bone adjacent to an implant, leading to better fixation. This has already been shown in several clinical studies, but awaits further evaluation.
